# Deep Neural Network-Evaluated Thermal Conductivity for Two-Phase WC-M (M = Ag, Co) Cemented Carbides

**DOI:** 10.3390/ma15186269

**Published:** 2022-09-09

**Authors:** Shiyi Wen, Xiaoguang Li, Bo Wang, Jing Tan, Yuling Liu, Jian Lv, Zhuopeng Tan, Lei Yin, Yong Du

**Affiliations:** 1 School of Metallurgy and Environment, Central South University, Changsha 410083, China; 2 Key Laboratory of Computing and Stochastic Mathematics (Ministry of Education), School of Mathematics and Statistics, Hunan Normal University, Changsha 410081, China; 3 State Key Laboratory of Powder Metallurgy, Central South University, Changsha 410083, China; 4 Institute of Engineering Research, Jiangxi University of Science and Technology, Ganzhou 341000, China; 5 Ganzhou Achteck Tool Technology Co., Ltd., Ganzhou 341000, China

**Keywords:** composite materials, cemented carbides, WC-Ag, WC-Co, thermal conductivity, Deep Neural Network

## Abstract

DNN (Deep Neural Network) is one kind of method for artificial intelligence, which has been applied in various fields including the exploration of material properties. In the present work, DNN, in combination with the 10-fold cross-validation, is applied to evaluate and predict the thermal conductivities for two-phase WC-M (M = Ag, Co) cemented carbides. Multi-layer DNNs were established by learning the measured thermal conductivities for the WC-Ag and WC-Co systems. It is observed that there are local-minimum regions for the loss functions during training and testing the DNNs, and the presently utilized Adam optimizer is valid for breaking the local-minimum regions. The good agreements between the DNN-evaluated thermal conductivities and the measured ones manifest that the DNNs were well trained and tested. Moreover, another 1000 input data points were randomly generated for the established DNNs to predict the thermal conductivities for WC-Ag and WC-Co systems, respectively. Compared with the thermal conductivities predicted by the previously developed physical model, the presently established DNNs show similarly robust predicting ability. Concerning the efficiency, it is demonstrated in the present work that machine learning is promising to explore the material properties, especially in the high-dimensional parameter space, more efficiently than previous models, and thus can considerably contribute to the corresponding material design with less time consumption and costs.

## 1. Introduction

Cemented carbide is one typical kind of composite materials, which consists of the hard phase and binder phase. It is of high hardness and toughness and is thus widely applied in the industry as inserts for metal cutting, rolls for hot/cold-roll and wear parts, etc. [1,2,3,4,5,6]. Cemented carbide mostly serves at high temperatures. In this situation, heat conduction is one key factor for preventing the concentration of thermal stress and thus prolonging the service life. However, the current focus for studying cemented carbides is the mechanical properties while the thermophysical properties are relatively less investigated, in which thermal conductivity is of great importance. High thermal conductivity can considerably prolong the service life of materials, especially for the cemented carbides which usually serve at high temperatures [6]. Therefore, it is necessary and urgent to study thermal conductivity for cemented carbides. In our previous work, a physically sound model for evaluating the thermal conductivity for composite materials was developed, which was applied to WC-M (M = Co, Ag) cemented carbides [7]. Although this model is of good reliability, it is critically dependent on the accuracy of the model parameters, i.e., ITR (Interfacial Thermal Resistance) and the thermal conductivity of pure WC phase, which are difficult to obtain [7]. We previously obtained both the model parameters by fitting the numerous thermal conductivities of WC-M (M = Co, Ag) cemented carbides [7], which is not efficient enough. To the best of our knowledge, the current efficient way to obtain the ITR and the thermal conductivity of pure WC phase are TDTR (Time-domain thermoreflectance) [8,9] and SThM (Scanning thermal microscopy) methods [10], but they can be hardly utilized to measure them at high temperatures, which are necessary for modeling the thermal conductivity for cemented carbides. Therefore, in order to avoid this difficulty and thus improve the evaluating efficiency, machine learning is an alternative approach which is not based on these model parameters due to its algorithm. It is believed that applying machine learning to evaluate the thermal conductivity for cemented carbides is of both scientific and technological interest.

Recently, machine learning methods have developed very fast due to the improvement of computer science and technology [11,12,13,14,15,16,17,18]. Deep learning, which is based on DNN (Deep Neural Network), is one of the most powerful methods [19,20,21,22,23,24,25]. DNN achieves great success in various applications such as image recognizing, natural language process, and biological technology. As a well-known case, Alpha-Go was trained by the multi-layer DNN and beat the best human player in the game of Go [26]. Nowadays, more and more researchers are applying DNN to evaluate the properties of materials [27,28,29,30,31] and obtained reasonable and desired results. However, it is well known that DNN can produce reasonable results, but the obtained model parameters are difficult to be explained physically. It is thus expected that if both the physical model and DNN are applied to study material properties, the obtained results can be more explainable and reliable by the cross-validation of the two methods. Therefore, it is also of great interests to make comprehensive comparisons between the physical model and DNN on the performance for assessing the material properties. It should be noted that for feeding DNN models, the reliability of data needs to be ensured. As we investigated before [7], the thermal conductivity data available in literature for two-phase WC-Co and WC-Ag cemented carbides are of great quantity and quality. Thus, in the present work, two-phase WC-Co and WC-Ag are chosen as the model systems. Based on the preceding descriptions, the aims of the present work are: (*i*) to develop DNN for evaluating the thermal conductivity for two-phase WC-Ag and WC-Co systems where 10-fold cross-validation is also applied in order to increase the evaluation efficiency and accuracy, (*ii*) to compare the DNN-evaluated thermal conductivities with the measured ones for verifying the reliability of the presently established DNN models, and (*iii*) to randomly produce 2000 groups of input data points which are to be fed into the established DNN models as well as the previously developed physical models for making comparisons, thus validating the ability of the presently established DNN models to predict thermal conductivity for WC-Ag and WC-Co cemented carbides.

## 2. Materials and Methods

### 2.1. Deep Neural Network

A DNN is a sequential alternative composition of linear functions and nonlinear activation functions. Given m,n≥1, let $\Theta \left(x\right)=Wx+b$ be a linear function mapping ${\mathbb{R}}^{n}\to {\mathbb{R}}^{m}$, where $W\in {\mathbb{R}}^{m\times n}$ and $b\in {\mathbb{R}}^{m}$ are called weight and bias, respectively. The non-linear activation function $\sigma \left(u\right):\mathbb{R}\to \mathbb{R}$. By applying the activation function component wisely, a DNN with L+1 layers can be expressed in an iterative compact way:

(1) $$\begin{array}{c} T\left(x\right)=T^{L}\left(x\right);\\ T^{l}\left(x\right)=\left[\Theta^{l}\circ \sigma \right]\left(T^{l-1}\left(x\right)\right),\;l=1,2,\ldots ,L, \end{array}$$

With $T^{0}\left(x\right)=\Theta^{0}\left(x\right)$, or equivalently,

(2) $$T\left(x\right)=\Theta^{L}\circ \sigma \circ \Theta^{L-1}\circ \sigma \cdots \circ \Theta^{1}\circ \sigma \circ \Theta^{0}\left(x\right).$$

**Figure 1:**
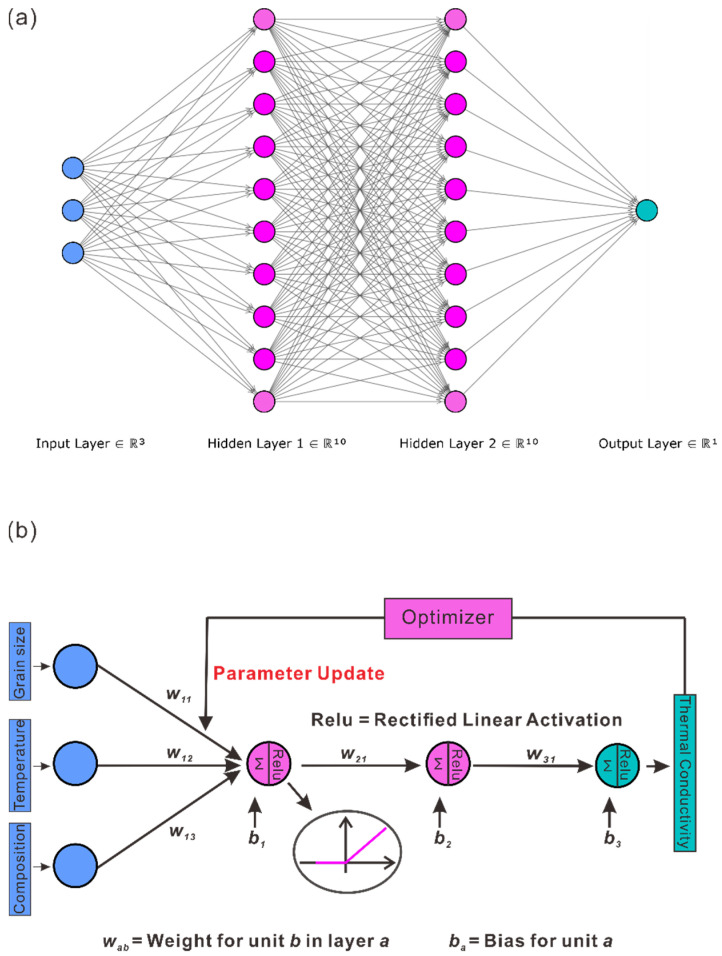
(**a**) The overall structure of the multi-layer DNN; (**b**) The forward propagation and backpropagation of the DNN.

Here, $\Theta^{l}\left(x\right)=W^{l}x+b^{l}:{\mathbb{R}}^{n^{l}}\to {\mathbb{R}}^{n^{l+1}}$. This DNN is also considered to have L hidden layers and its l-th layer has $n^{l}$ neurons. Denoting θ as all the weights and biases, we write a DNN as $T\left(x;\theta \right)$. In Figure 1a, we illustrate a DNN with 2 hidden layers. The input dimension is 3 while the output dimension is 1. There are 10 neurons in each hidden layer.

DNN has the property of universal approximation [32], which means a sufficiently large DNN can approximate any continuous function. The thermal conductivity of WC-M cemented carbides can be determined by the grain size of WC, the temperature, and the volume fraction of WC phase [7]. There is no doubt that we can depend on the universal approximation property of DNN to investigate such a complicated quantitative relation between the thermal conductivity and WC grain size, temperature, and WC volume fraction. It should be noted that DNN has the ability to analyze the feature importance automatically, and thus it is not necessary to pretreat the features.

### 2.2. DNN-Based Thermal Conductivity Model

We modeled the thermal conductivity as a DNN $y=T\left(x;\theta \right)$, where $x\in {\mathbb{R}}^{3}$ corresponds to the grain size of WC (m), the temperature (K), and the volume fraction of WC phase, while y∈ℝ is the thermal conductivity(W/mK). Given x, the predicted thermal conductivity is $\hat{y}=T\left(x;\theta \right).$ When choosing the depth and width of the DNN model, it is worth noting that the deeper and wider the DNN is, the more powerful the DNN is, but the computational cost is also larger. To balance the model capacity and the computation cost, in this work, we chose a DNN with 2 hidden layers. The first layer contains 128 neurons while the second layer contains 10 neurons. The activation function is chosen to be ReLU (Rectified Linear Activation) function $\sigma \left(u\right)=\max\left\{0,x\right\}$, which is one of the most popular activation functions and helps to overcome the gradient vanishing problem [21].

The weights and biases θ in DNN should be trained by experimental data to achieve the best prediction. Given a training data set ${\left\{\left(x_{i},y_{i}\right)\right\}}_{i=1}^{n}$, we find the best θ by minimizing the MSE (Mean Square Error) of the predictions ${\hat{y}}_{i}=T\left(x_{i};\theta \right)$, where MSE is defined as

(3) $$MSE\left(\theta \right)=\frac{1}{n}\overset{n}{\underset{i=1}{\sum }}{\left|y_{i}-{\hat{y}}_{i}\right|}^{2}=\frac{1}{n}\overset{n}{\underset{i=1}{\sum }}{\left|y_{i}-T\left(x_{i};\theta \right)\right|}^{2}.$$

This minimization is done by the gradient-based optimization algorithms. The DNN can be evaluated efficiently by forward propagation algorithm, while the gradient is calculated by backward propagation algorithm [19]. This procedure is shown in Figure 1b. We choose Adam [33] with mini batch as the optimization algorithm, which is one of the most popular algorithms in deep learning committee. The learning rate is chosen to be 0.001, while other parameters are set as default.

In machine learning, we need to divide our data set into training data and test data. The training data are used to train the model (i.e., to determine parameters θ) while test data are used to measure the generalization ability of the model, which mean whether the trained model is still valid for data out of training data.

**Figure 2:**
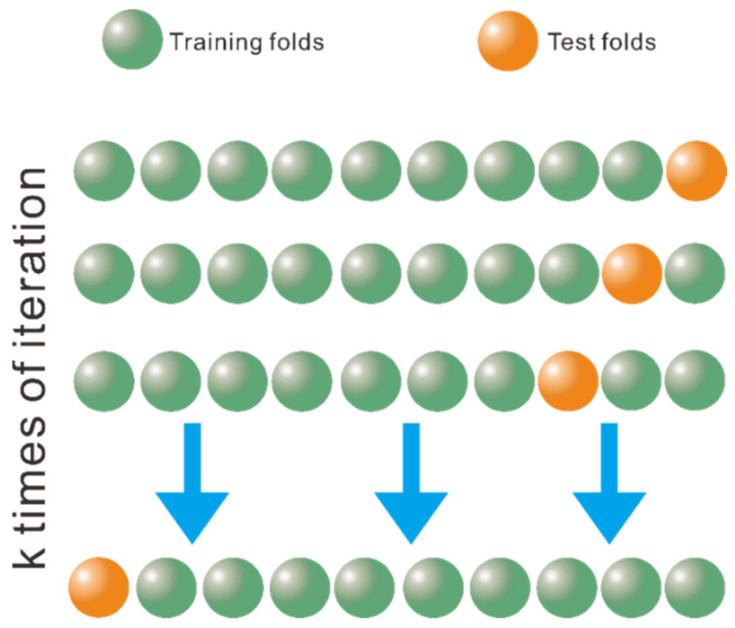
The scheme of the k-fold cross-validation, which divides the dataset into training folds and test folds for k times of learning iterations.

To reduce the bias introduced by data splitting and improve the accuracy of the obtained DNN, K-fold cross validation is a very useful technique. K-fold cross validation randomly divides the entire dataset into K parts. In each fold, K-1 parts are used as training data and one part is left as test data. The average performance of the K folds measures the generalization ability of DNN model. Generally, K is set to be 5 or 10. In the present work, 10-fold cross validation is used in all the numerical experiments. An illustration of 10-fold cross validation procedure is shown in Figure 2.

## 3. Results and Discussion

### 3.1. DNN Model for WC-Ag System

**Figure 3:**
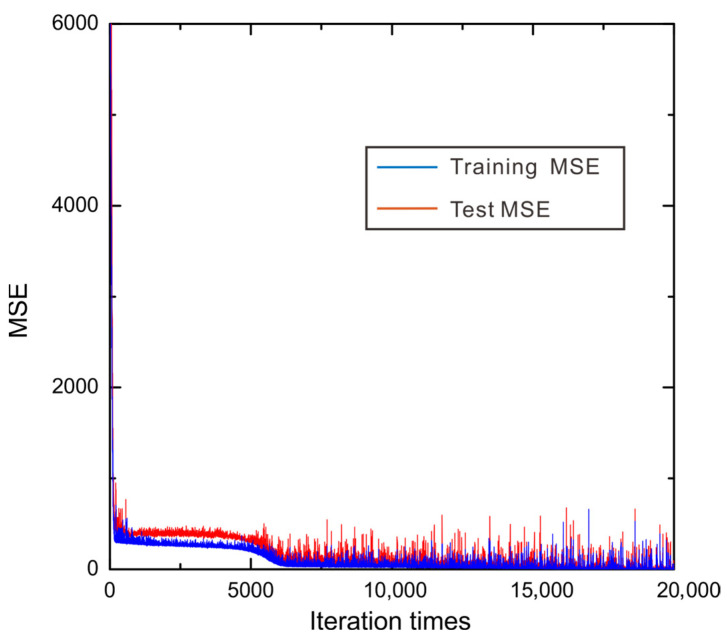
The training and validating MSEs varying with the iteration times for evaluating the thermal conductivity for the WC-Ag system.

We collected data on the thermal conductivity and corresponding grain size of WC phase, temperature, and volume fraction of WC phase for two-phase WC-Ag system from Ref. [34]. In Ref. [34], the two-phase structure of WC-Ag system can be identified. Meanwhile, there are 90 lines of thermal conductivity data, where 81 lines are training data and 9 lines are test data. In order to train such a small dataset, 10-fold cross validation is applied to split these data into training and test datasets. We trained each fold by 20,000 iteration times with batch size 10. The whole process of training and test took 58 min using the personal computer (CPU: Intel(R) Core (TM) i5-6300HQ). Table 1 shows the training and test *R*^2^ score of each fold. From Table 1, we can find that DNN performs very well on both training and test dataset, since the training and test $R^{2}$ are larger than 0.95 for all folds. The average training and test $R^{2}$ are, respectively, 0.9859 and 0.9745, which suggest that the evaluation and prediction of the presently obtained DNN models are reliable. It can be also seen that fold 3 demonstrates the best training and test *R*^2^ score among all the folds. Therefore, fold 3 is considered to be the most appropriate DNN model for the next thermal conductivity evaluations for the two-phase WC-Ag system. In order to indicate the whole evaluation process by DNN model, Figure 3 is plotted to show the values of training MSE and test MSE for fold 3 during the entire machine-learning process. Note that the MSE for training demonstrates the performance for fitting the DNN model while the MSE for test presents the generalization ability of the obtained DNN model. It can be seen from Figure 3 that both the training and test MSEs decrease with the iteration time, and the MSEs of the training dataset is generally smaller than those of the test datasets. In order to obtain the best DNN model, both the training and test MSE should be minimized globally or near globally, meaning that the local minimum should be avoided. However, it can be seen from Figure 3 that the curve is of a ladder-like shape, indicating that in the ‘ladder’, the loss function falls into the local minimum. In such a situation, Adam optimizer [33] is utilized to break this local minimum successfully. Moreover, the training and test MSEs are small enough that they cannot further decrease after 10,000 iteration times. It should be noted that machine-learning performance of other folds are similar to fold 3.

**Figure 4:**
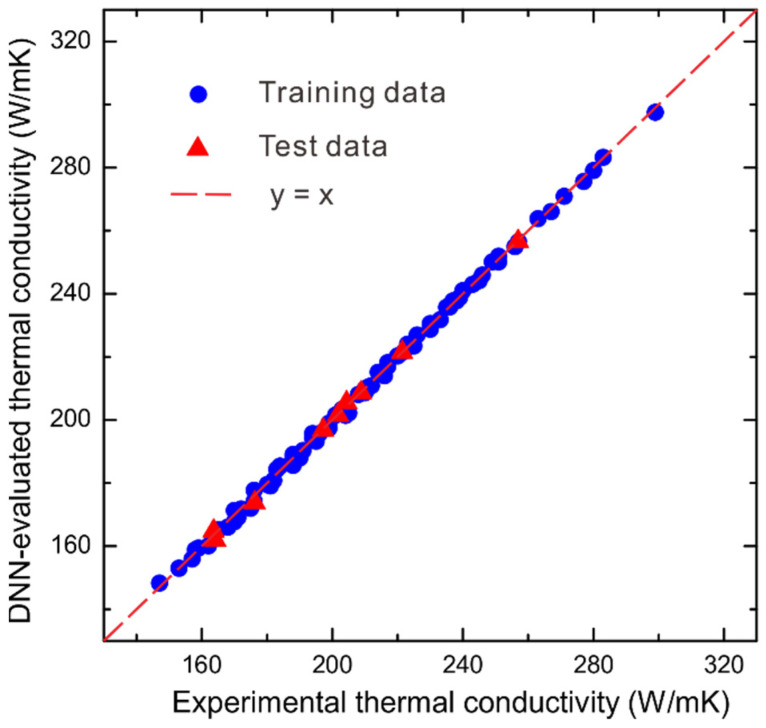
The comparisons of training and test thermal conductivities by fold 3 with the measured ones for WC-Ag system.

Subsequently, using the well-established DNN model of fold 3, the thermal conductivities are evaluated and compared with the measured ones. Figure 4 shows the comparisons of training and test thermal conductivities with the measured ones, respectively, where we can see that both the training and test thermal conductivities by fold 3 show quite good agreements with the measured ones.

### 3.2. DNN Model for WC-Co System

**Figure 5:**
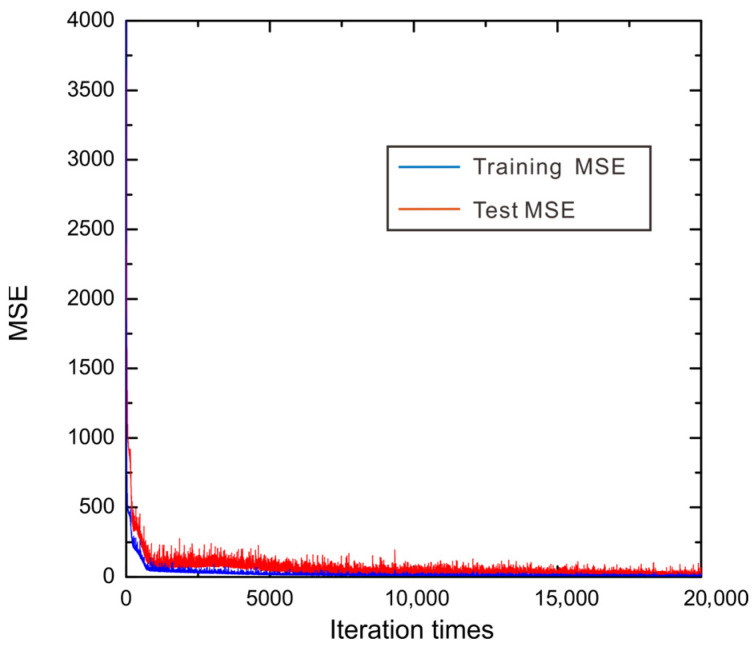
The training and test MSE varying with the iteration time for evaluating the thermal conductivity for WC-Co system.

In our previous work, it was found that the thermal conductivity for two-phase WC-Co system abruptly changes at 695 K due to the phase transition of the binder phase Co [7]. It should be noted that in Ref. [7], XRD (X-ray diffraction) and SEM (Scanning Electron Microscope) were conducted, which can verify the two-phase structure of prepared WC-Co samples. Therefore, in order to obtain reasonable calculation results by physical models, we operated the calculations of the thermal conductivity for WC-Co system above and below 695 K separately in our previous work [7], while in this work we are trying to use the robust DNN method to train all the thermal conductivity data in one operation, regardless of the phase-transition temperature of Co. The training and test thermal conductivity datasets of thermal conductivities for WC-Co system are taken from Refs. [7,35]. In total, 109 lines of thermal conductivities of WC-Co system are collected, in which 98 lines are the ones for training while 11 lines are for test. The value of iterations of training and test for the neuron network is also set to be 20,000. Similarly, in one iteration, 10 rounds of training and test are conducted by the 10-fold cross-validation, as described in Section 2. Table 2 presents the training and test *R*^2^ score of each fold. The average *R*^2^ scores for training and test are 0.9838 and 0.9449, respectively, indicating that the 10-fold cross validation performs well. It can be also seen from Table 2 that fold 3 shows the best *R*^2^ score and thus it is utilized for the next evaluations. Since the main purpose is to minimize the MSE, the variation of training and test MSE with the iteration times for fold 3 is plotted in Figure 5. It can be seen from Figure 5 that the training and test MSE decrease with the iteration time, indicating that the presently determined DNN structure for evaluating thermal conductivity for two-phase WC-Co system is reasonable. By using the Adam optimizer, the local minimum can also be broken successfully to continue the minimization process, as presented in Figure 5. After 5000 iteration time, the MSE value remains almost the same and cannot further decrease. Therefore, the deep-learning process is considered to be finalized. The whole process for training and test took 67 min using the personal computer (CPU: Intel(R) Core (TM) i5-6300HQ).

**Figure 6:**
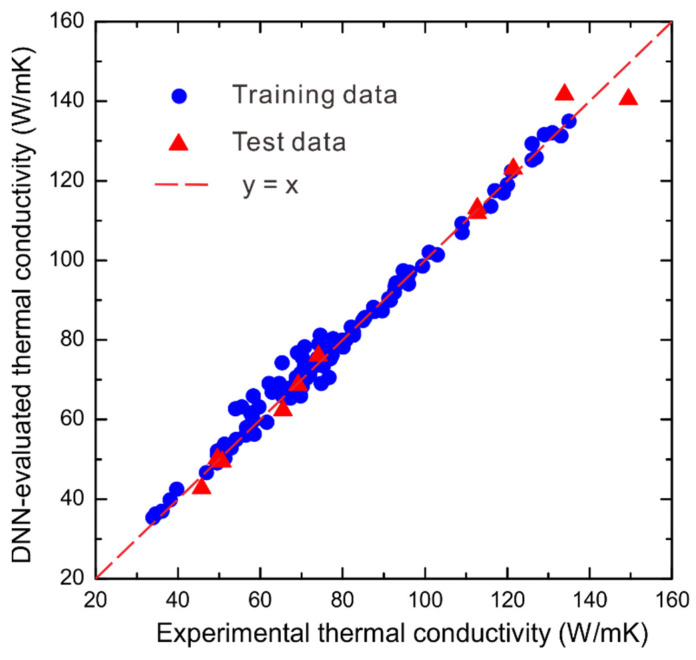
The comparison between the training and test thermal conductivities by fold 3 with measured thermal conductivities for the WC-Co system.

Applying the obtained DNN model by fold 3, the thermal conductivities for WC-Co system are evaluated and compared with the measured ones from our previous work [7] and Ref. [35]. As shown in Figure 6, all the training and test thermal conductivities agree quite well with the measured ones, demonstrating that the obtained DNN model by fold 3 is reasonable. It should be noted that although the presently established DNN model can better reproduce the measured thermal conductivities than the physical model [7], a total of hundreds of parameters were utilized in this DNN model, which are much more than the parameters used in the physical model (6 parameters). Next, we will make a comprehensive comparison between this established DNN model and physical model to further validate the prediction performance of the present DNN model.

To be concluded, the DNNs for evaluating thermal conductivity for both two-phase WC-Co and WC-Ag systems have been established. The generalizability of the established DNNs is also of great interest. Thus, in future work, we will be able to develop one general DNN to evaluate thermal conductivity for all WC-based cemented carbides with more thermal conductivities being measured.

### 3.3. Validation of the Present DNN Models

**Figure 7:**
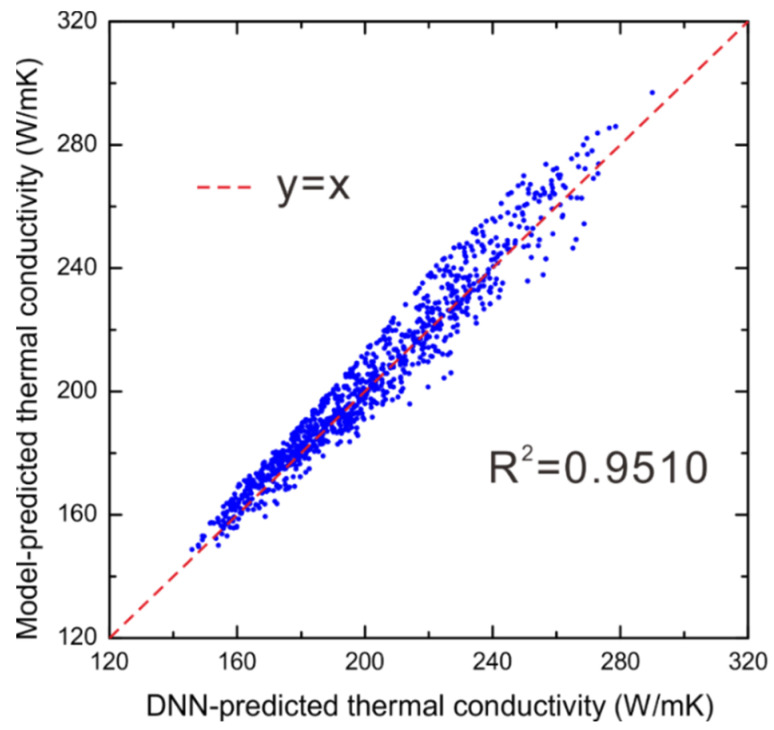
The comparison between the DNN-predicted thermal conductivities and the evaluated ones by the physical model for the WC-Ag system.

It is of great interest to validate the performance of the presently established DNN model for predicting the thermal conductivities for WC-Ag and WC-Co systems, in comparison with the physical model. Therefore, another 1000 input data points at random phase compositions, grain sizes and temperatures were generated for WC-Ag and WC-Co systems, respectively. We selected fold 3 for predicting thermal conductivities of both WC-Ag and WC-Co systems since they performed best among all the folds. For WC-Ag system, Figure 7 is plotted to show the comparison between the DNN-predicted thermal conductivities and the ones evaluated by the physical model [7], where the *R*^2^ value is determined to be 0.9510. The good agreement indicates that this obtained DNN model also achieves a good performance for predicting the thermal conductivities for WC-Ag system.

**Figure 8:**
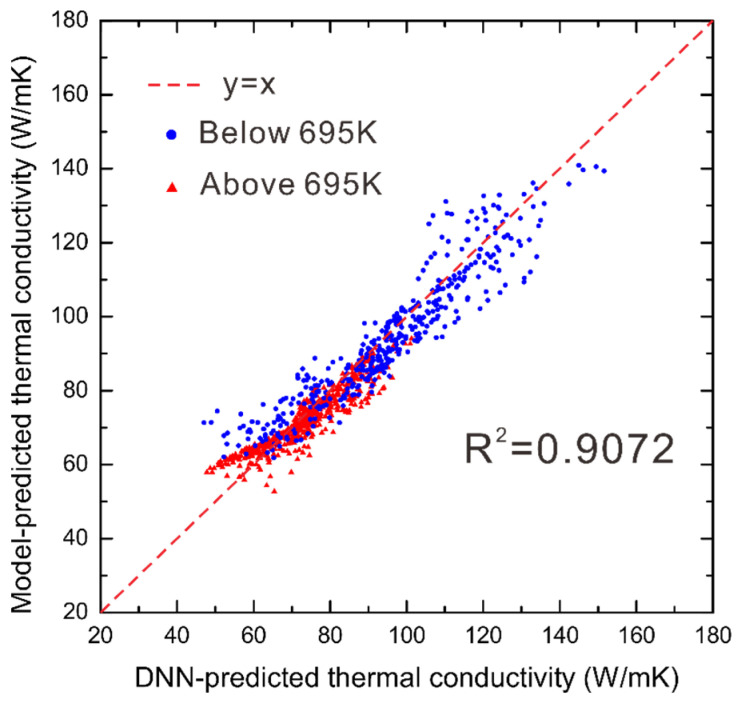
The comparison between the DNN-predicted thermal conductivities and the evaluated ones by the physical model for the WC-Co system.

Moreover, Figure 8 presents the comparison between the DNN-predicted thermal conductivities and the ones evaluated by our previously established physical model [7]. The thermal conductivities are separated into two datasets, i.e., above 695 K and below 695 K. It can be found that although we established only one DNN model regardless of the phase-transition temperature of the binder phase Co, the DNN-predicted results agree well with the ones by the physical models [7], with the *R*^2^ score as 0.9072. Therefore, the presently established DNN model for evaluating the thermal conductivity for WC-Co system is considered to have a similarly good prediction ability to the physical models [7]. It is worth mentioning that such a good prediction ability is partly due to the strategy of the 10-fold cross validation, where the test dataset contributes a lot to improving the prediction ability of the DNN model.

In comparison with developing the physical model, establishing DNN models has its advantages and disadvantages. From the time consumption for establishing the DNN models we can see that it is more efficient than obtaining the physical model. However, the number of parameters utilized in each DNN model is much more than the physical model. Furthermore, if more factors should be considered in the physical model for evaluating the thermal conductivity for composite materials such as the porosity, grain distribution and shape factor, etc., the difficulty for developing the physical model will be increased exponentially. For instance, in our previous work [7], to evaluate only one of the key factors (i.e., ITR for thermal conductivity) was complex enough and thus was very time-consuming. In this case, DNN models are promising to improve this situation to consider other factors for assessing the material properties with less time consumptions and costs, and thus can further contribute to design materials with desired properties efficiently.

## 4. Conclusions

In the present work, the DNN method, in combination with the 10-fold cross validation, was applied to evaluate and predict the thermal conductivities for two-phase WC-Ag and WC-Co systems. The main conclusions are as follows:

1. The established DNN models can well reproduce the measured thermal conductivities for the WC-Ag and WC-Co systems, presenting their good performances for the evaluations;
2. The present DNN models demonstrate good performances for predicting the thermal conductivities for WC-Ag and WC-Co systems since the DNN-predicted thermal conductivities agree well with the ones predicted by the physical model;
3. Even though the number of parameters utilized in the DNN models is much more than the that in the physical model, the DNNs are of higher efficiency and thus are promising to be utilized in more complex explorations for material properties.

## Figures and Tables

**Table 1:** The training and test $R^{2}$ scores of 10-fold cross validation for evaluating thermal conductivity for WC-Ag system. The average $R^{2}$ is shown in the last column.

Fold	1	2	3	4	5	6	7	8	9	10	Average
Training $R^{2}$	0.9836	0.9675	0.9988	0.9893	0.9982	0.9667	0.9880	0.9960	0.9810	0.9903	0.9859
Test $R^{2}$	0.9727	0.9502	0.9987	0.9884	0.9968	0.9175	0.9615	0.9878	0.9855	0.9861	0.9745

**Table 2:** The training and test $R^{2}$ scores of 10-fold cross validation for evaluating thermal conductivity for WC-Co system. The average $R^{2}$ is shown in the last column.

Fold	1	2	3	4	5	6	7	8	9	10	Average
Training $R^{2}$	0.9916	0.9772	0.9856	0.9735	0.9862	0.9890	0.9834	0.9849	0.9780	0.9885	0.9838
Test $R^{2}$	0.9400	0.8548	0.9881	0.9364	0.9775	0.9243	0.9653	0.9623	0.9351	0.9653	0.9449

## Data Availability

The data presented in this study are available on request from the corresponding author.
